# Veno-arterial CO_2_ content gradient and veno-arterial CO_2_ to arterial-venous O_2_ content ratio for outcome prediction after pediatric cardiac surgery: a prospective study

**DOI:** 10.1186/s40635-025-00834-9

**Published:** 2025-12-10

**Authors:** Vladimir L. Cousin, Raphael Joye, Tomasz Nalecz, Tornike Sologashvili, Maurice Beghetti, Cyril Jaksic, Julie Wacker, Angelo Polito

**Affiliations:** 1 Paediatric Intensive Care, Neonatal Medicine and Emergency, AP-HP Paris Saclay University, Bicêtre Hospital, 78, rue du Général Leclerc, 94270 Le Kremlin-Bicêtre, France; 2 Paediatric Intensive Care Unit, Department of Paediatrics, Gynecology and Obstetrics, Geneva University Hospital, Geneva University of Medicine, Geneva, Switzerland; 3 Paediatric Cardiology Unit, Department of Paediatrics, Gynecology and Obstetrics, Geneva University Hospital, Geneva University of Medicine, Geneva, Switzerland; 4 The Labatt Family Heart Centre, Division of Cardiology, The Hospital for Sick Children, Department of Paediatrics, University of Toronto, Toronto, Canada; 5 Paediatric Cardiac Surgery Unit, Surgery Department, Geneva University Hospital, Geneva University of Medicine, Geneva, Switzerland; 6 Division of Clinical Epidemiology, Geneva University Hospital, Geneva University of Medicine, Geneva, Switzerland

**Keywords:** Cardiopulmonary bypass, Congenital heart disease, Postoperative care, Veno-arterial CO_2_ content gradient, Venous-arterial CO_2_ to arterial-venous O_2_ content difference, Oxygen extraction, Lactate

## Abstract

**Introduction:**

CO_2_-derived variables, veno-arterial CO_2_ content gradient (ΔCCO_2_) and the ratio of ΔCCO_2_ with arterio-venous oxygen difference (AV-DO_2_) (ΔCCO2/AV-DO_2_), may have a potential role as indicators of low cardiac output and anaerobic metabolism, respectively. We sought to describe and evaluate the association of CO_2_-derived variables with patients’ outcomes in the post cardiopulmonary bypass (CPB) period in children.

**Methods:**

Prospective, single-center, study enrolling children post-CPB with paired arterial and venous blood gases for determination of lactate, O_2_ extraction, ΔCCO_2_, and ΔCCO_2_/AV-DO_2_ at admission (H0), and at 6 (H6), 12 (H12) and 24 (H24) hours. Different clinical patterns were defined based on the presence of an anaerobic context or a hypoperfusion context, using both O_2_ and CO_2_-derived variables. The presence of anaerobic metabolism was defined with a lactate > 2 mmol/l and ΔCCO_2_/AV-DO_2_ > 1.8; the presence of hypoperfusion was defined with an O_2_ extraction > 30% and ΔCCO_2_ > 6 mL. The potential association of duration of amine support and mechanical ventilation was tested with CO_2_-derived variables and specific clinical patterns.

**Results:**

A total of 51 patients with a median age of 36 (IQR 11–85) months were included. Median admission ΔCCO_2_ was 9.3 mL (IQR 5.6–11.4) with 72% above 6 mL. Median ΔCCO_2_/AV-DO_2_ was 2.1 (IQR 1.5–2.4) with 58% above 1.8. Admission ΔCCO_2_ showed a significant association with the duration of mechanical ventilation (R2 21.6, *p* value = 0.001) but not with the duration of vasoactive support. Neither H0 ΔCCO_2_ nor H0 ΔCCO_2_/AV-DO_2_ improved outcome prediction by a model including lactate and O_2_ extraction. Anaerobic metabolism context showed a significant association with prolonged vasoactive support [28.4 (CI 95% 12.2–44.6) *p* = 0.001] and mechanical ventilation duration [1.4 (95% CI 0.62–2.3) *p* = 0.003]. In hypoperfusion context, neither duration of vasoactive support nor mechanical ventilation appeared different in the subgroups analysis.

**Conclusion:**

CO_2_-derived variables may improve outcome prediction after cardiac surgery in pediatric patients. Further evaluation in larger multicentered trials is necessary to improve its validation.

**Supplementary Information:**

The online version contains supplementary material available at 10.1186/s40635-025-00834-9.

## Introduction

Evaluation of oxygen (O_2_) metabolism is frequently used in the pediatric intensive care unit (PICU) to assess patients’ hemodynamic status [1–3]. Lactate level and O_2_ extraction are the main variables used in this setting [4]. However, both may experience limitations in clinical settings. Falsely elevated lactate can be caused by hyperglycemia or aminergic support with stimulation of beta-2 receptors while reduced clearance can be experienced in cases of renal or liver failure [5]. O_2_ extraction is affected by multiple variables, including cardiac output, arterial O_2_ saturation, hemoglobin level and O_2_ consumption [3]. Moreover, cases of falsely normal extraction have been reported, especially in the presence of sepsis [5, 6]. Carbon dioxide (CO_2_) metabolism has been proposed as an additional tool to assess hemodynamic status [6–8]. The main variables described are the veno-arterial CO_2_ content gradient (ΔCCO_2_) and the ratio of ΔCCO_2_ with arterio-venous oxygen difference (AV-DO_2_) (ΔCCO_2_/AV-DO_2_).

Derived from the Fick equation, ΔCCO_2_ reflects the adequacy of the balance between the cardiac output and the CO_2_ production (VCO_2_), according to the formula ΔCCO_2_ = VCO_2_/cardiac output [6]. An increase in ΔCCO_2_ is then directly related to a decrease in cardiac output if the VCO_2_ is stable [9, 10]. Even in the case of anaerobic metabolism, a production of CO_2_ occurs through buffering of bicarbonate [HCO_3_^−^] by acidic cations [H^+^], maintaining a certain level of VCO_2_, while the cardiac output is severely reduced, leading to an accumulation of CO_2_ in venous blood. Elevation of ΔCCO_2_ above 6 mL is related to worse outcomes in septic shock and other ICU admissions [4, 11]. The ratio of ΔCCO_2_/AV-DO_2_ has been less studied and acts as a surrogate for the respiratory quotient. It evaluates the balance between CO_2_ production and O_2_ consumption, both determined according to the Fick equation: the ΔCCO_2_ estimates VCO_2_ and the AV-DO_2_ estimates VO_2_. Under physiological conditions, VCO_2_ should not exceed VO_2_, and, therefore, a ratio > 1 should be encountered only in anaerobic situations when the anaerobic VCO_2_ exceeds the decreased VO_2_ [12]. In septic shock, elevated ΔCCO_2_/AV-DO_2_ with values ranging between 1.2 and 1.6 have been related to worse outcomes, even in cases of normal lactate levels [13, 14] 

Studies evaluating both variables and their use in clinical practice remain rare in the pediatric population, particularly for ΔCCO_2_/AV-DO_2_. Despite differences in study populations and outcomes definitions, ΔCCO_2_ has been associated with different outcomes after pediatric cardiac surgery, ranging from increased mortality, prolonged mechanical ventilation or worse composite outcomes including ECMO requirement or high vasopressor requirement [15–17]. Of particular significance, CO_2_-derived variables are not subject to the limitations associated with lactate and O_2_-extraction measurements [18]. Furthermore, integrating data from both O_2_ and CO_2_ variables could prove valuable in identifying distinct clinical patterns, according to the presence of anaerobic metabolism or a state of hypoperfusion, with potentially different trajectories in the PICU. This approach mirrors findings in septic shock patients, where these indices have been used to detect low cardiac output and inadequate oxygen delivery situations, with significantly different outcomes even in the presence of similar O_2_-derived variables [14, 19–22].

We hypothesized that the use of CO_2_-derived variables could improve hemodynamic assessment after surgery for congenital heart disease (CHD). Our study sought to evaluate the capacity of ΔCCO_2_ and ΔCCO_2_/AV-DO_2_ to predict outcomes after cardiac surgery in pediatric patients.

## Methods

### Study design and population

This prospective study was conducted between November 2022 and December 2023 at the PICU of Geneva University Hospitals. The study, untitled “Cardiac output monitoring using CO2 metabolism in children after cardiac surgery” was registered and approved by the local ethic committee (Commission Cantonale d’Ethique et de la Recherche de l’Etat de Genève) on the 27th of September 2022, project number CCER 2022-00488. This study adheres to national regulations on medical research and international guidelines of the Helsinki Declaration. Informed consent was obtained from each subject or the subject’s legally authorized representative.

The study population consisted of children (0–18 years) admitted after cardiopulmonary bypass (CPB) for CHD surgery. Patients were excluded if they did not have both a central venous catheter and an arterial catheter placed before the surgery, if they required extracorporeal membrane oxygenation or if the chest was left open upon leaving the operating room (*N* = 1).

The primary outcome was the duration of vasoactive support. The secondary outcome was the duration of mechanical ventilation. We also sought to evaluate whether CO_2_-derived variables improve outcome prediction beyond O_2_-derived variables (lactate and O_2_ extraction) and, when integrated with them, enable refined definitions of hemodynamic clinical patterns.

### Data collection

Demographic and clinical variables collected were: age, weight, cardiac defect, surgery risk evaluation using the Risk Adjustment for Congenital Heart Surgery (RACHS) score [23], CPB and aortic cross-clamp time. Data were prospectively collected at the following 4 time points: at PICU admission (H0) following cardiac surgery and after 6 h (H6), 12 h (H12) and 24 h (H24). At every time point, vasoactive support was reported using the Vaso-Inotropic Score (VIS) [24], and paired arterial and central venous blood gases were collected with the following data: pH,O_2_ saturation, partial pressure in O_2_, partial pressure in CO_2_, hemoglobin level (g/dL), temperature, inspired O_2_ fraction, lactate (mmol/L). O_2_- and CO_2_-derived variables were then calculated.

Blood gases were performed using a Radiometer ABL-800 (Radiometer, Copenhagen, Denmark).

O_2_-derived variables were calculated as follows:

O_2_ extraction = arterial O_2_ saturation − venous O_2_ saturation.

Other O_2_-derived perfusion markers were directly available on the blood gases: arterial lactate (mmol/L) and superior vena cava oxygen saturation [SVO_2_ (%)].

CO_2_-derived variables were calculated according to Douglas’ formula [25] (detailed in supplementary materials):

$$\Delta {\text{CC}}{{\text{O}}_{2}} = {\text{CvC}}{{\text{O}}_{2}} - {\text{CaC}}{{\text{O}}_{2}}.$$

ΔCCO_2_/AV-DO_2_ calculation:

Calculation for oxygen content in arterial (CaO_2_) or venous (CvO_2_) blood were determined as follows:

$$\begin{aligned} {\text{Ca}}{{\text{O}}_{2}} & = {\text{ Hb }} \times { 1}.{34 } \times {\text{ Sa}}{{\text{O}}_{2}} + \, 0.00{3 } \times {\text{ Pa}}{{\text{O}}_{2}}\;{\text{and}}\\ {\text{Cv}}{{\text{O}}_{2}} & = {\text{ Hb }} \times { 1}.{34 } \times {\text{ ScV}}{{\text{O}}_{2}} + \, 0.00{3 } \times {\text{ Pa}}{{\text{O}}_{2}}. \end{aligned}$$

### Definition of anaerobic metabolism and hypoperfusion subgroups

Two distinct hemodynamic clinical patterns were defined based on O_2_− and CO₂- derived variables, as incorporating CO₂ parameters alongside lactate and O_2_-extraction may refine outcome predictions in the PICU.

To assess *anaerobic metabolism*, we categorized patients using a combination of lactate levels and the ΔCCO₂/AV-DO₂. Since lactate reflects tissue hypoxia and impaired oxidative metabolism, and ΔCCO₂/AV-DO₂ serves as an index of respiratory quotient changes in low-flow states, their integration might enhance the identification of anaerobic metabolism. Four subgroups were defined:

- *Group 1*: lactate ≤ 2 mmol/L and ΔCCO₂/AV-DO₂ ≤ 1.8 (normal metabolism)
- *Group 2*: lactate > 2 mmol/L and ΔCCO₂/AV-DO₂ ≤ 1.8 (isolated hyperlactatemia without anaerobic CO_2_ production)
- *Group 3*: lactate ≤ 2 mmol/L and ΔCCO₂/AV-DO₂ > 1.8 (CO₂ anaerobic production despite normal lactate)
- *Group 4*: lactate > 2 mmol/L and ΔCCO₂/AV-DO₂ > 1.8 (both metabolic acidosis and anaerobic CO_2_ production, suggesting potential severe anaerobic metabolism)

To evaluate *hypoperfusion*, we combined O_2_ extraction with ΔCCO₂, given that O_2_ extraction reflects the balance between O_2_ delivery and consumption, while ΔCCO₂ increases in the presence of stagnant or low-flow states.

- *Group 1*: O_2_ extraction ≤ 30% and ΔCCO₂ ≤ 6 mL (normal perfusion)
- *Group 2*: O_2_ extraction > 30% and ΔCCO₂ ≤ 6 mL (high oxygen demand with preserved CO₂ clearance)
- *Group 3*: O_2_ extraction ≤ 30% and ΔCCO₂ > 6 mL (low oxygen extraction with CO₂ stagnation, suggesting circulatory inefficiency)
- *Group 4*: O_2_ extraction > 30% and ΔCCO₂ > 6 mL (both high oxygen demand and impaired CO₂ clearance, indicating significant hypoperfusion).

### Statistical analysis

Categorical variables were expressed as proportions (%) and continuous variables as median and interquartile range (IQR) due to the non-normal distribution of variables. Elevated ΔCCO_2_ and ΔCCO_2_/AV-DO_2_ were defined according to published cutoffs: >6 mL for ΔCCO_2_ and >1.8 for ΔCCO_2_/AV-DO_2_ [7, 12, 26, 27]. Associations between ΔCCO_2_, ΔCCO_2_/AV-DO_2_; anaerobic or hypoperfusion subgroups at admission and clinical PICU outcomes were evaluated using linear regression. In order to assess the extent to which each of the different parameters (ΔCCO_2_ gradient, ΔCCO_2_/AV-DO_2_ or a ratio of ΔCCO_2_ or ΔCCO_2_/AV-DO_2_ between H0 and H6 or H24) improves the explained proportion of variance of different outcomes on top of what both lactate and O_2_-extraction already explain, multiple linear regressions were conducted. For each parameter, the R2 of both models with and without the parameter of interest was compared. A small increase in R2 was interpreted as a poor improvement in explaining the outcome. All models included both changes in lactate and O_2_-extraction as predictors and the outcomes were the duration of vasopressor support and the duration of mechanical ventilation. Standard errors were estimated using the Huber–White (HC3) robust method. *P* value < 0.05 was considered statistically significant and statistical analysis was performed using R version 4.2.2 and Stat software v.18 (STATA, College Station, Texas USA).

## Results

### Patients

A total of 51 patients were included. Patient characteristics and clinical outcomes are summarized in Table 1. The median duration of PICU stay was 2 days (IQR 2–3). No patient required emergent chest reopening or ECMO and there were no in-hospital deaths during the study period.

**Table 1:** Characteristics of patients at baseline

Variable	Median (IQR) or *N* (%)
Age (months)	36 (11–85)
Weight (kg)	13.7 (9.9–28)
RACHS categories	
1	5 (9.8)
2	12 (23.5)
3	20 (39.2)
4	6 (11.8)
6	1 (1.9)
Not classified	7 (13.8)
Duration of CPB (min)	70 (52–139)
Duration of aortic cross-clamp (min)	48 (32–90)
Admission lactate (mmol/L)	1.6 (1–2.1)
Admission O_2_ extraction (%)	29 (25–33)
Admission VIS	11 (5–22.5)
Aminergic support (hours)	17 (5–24.5)
Mechanical ventilation (days)	0 (0–1)
PICU stay duration (days)	2 (2–3)

*CBP* cardiopulmonary bypass, *IQR* interquartile range, *PICU* pediatric intensive care unit, *RACHS* risk adjustment for congenital heart surgery

### CO_2_ and O_2_-derived variables

**Fig. 1:**
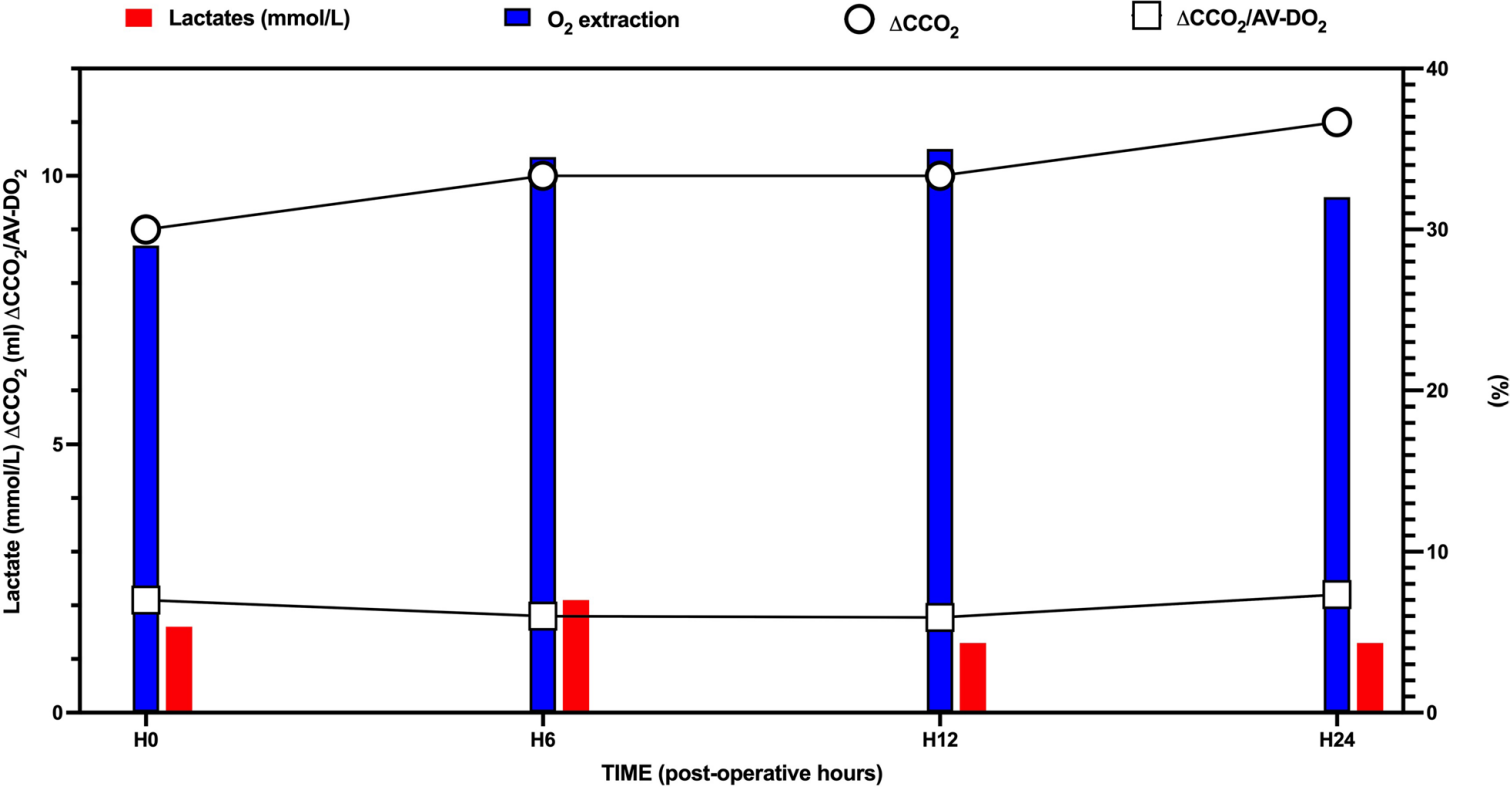
Evolution of O_2_ and CO_2_ variables over the first day of PICU stay post cardiopulmonary bypass. Evolution of O_2_-derived variables [lactate (mmol/L) and O_2_ extraction (%)] as well as CO_2_-derived variables (ΔCCO_2_ (mL) and ΔCCO_2_/AV-DO_2_) in the first 24 h of PICU after the cardiac surgery. Time points are as follows: admission H0, then at 6 h (H6), 12 h (H12) and 24 h (H24) of admission

At PICU admission, median ΔCCO_2_ was 9.3 mL (IQR 5.6–11.4) and median ΔCCO_2_/AV-DO_2_ was 2.1 (IQR 1.5–2.4). The evolution of CO_2_ and O_2_-derived variables over the first 24 h is depicted in Fig. 1. A gradual increase in ΔCCO_2_ and ΔCCO_2_/AV-DO_2_ was observed during this period, whereas lactate levels and O_2_ extraction tended to decrease after H12. At admission, 36 out of 50 patients (72%) had a ΔCCO_2_ > 6 mL and 29 out of 50 patients (58%) had a ΔCCO_2_/AV-DO_2_ > 1.8. The proportion of patients with elevated CO_2_-derived variables over the first 24 h after admission is detailed in Supplementary Fig. 1.

### Admission ΔCCO_2_, ΔCCO_2_/AV-DO_2_ and association with clinical outcomes

No significant association was identified between ΔCCO_2_ and the primary outcome. Univariate analysis showed a significant association between admission ΔCCO_2_ and duration of mechanical ventilation (R2 21.6, *p* value = 0.001) (Table 2). None of the outcomes were significantly associated with ΔCCO_2_/AV-DO_2_ at PICU admission (Table 2). Elevated ΔCCO_2_ (>6 mL) and ΔCCO_2_/AV-DO_2_ (>1.8) failed to significantly predict patients’ outcomes; only VA-CCO_2_/AV-O2 ratio > 1.8 approached significance for duration of mechanical ventilation (Supplementary Table 1).

**Table 2:** CO_2_-derived variables and their association with clinical outcomes

Variable	Coefficient (95% CI)	R2	*P* value
ΔCCO_2_ H0			
Vasoactive support (h)	0.93 (−0.64–2.5)	2.8	0.24
Mechanical ventilation (d)	0.14 (0.06–0.21)	21.6	0.001
ΔCCO_2_/AV-DO_2_ H0			
Vasoactive support (h)	2.87 (−6.1–11.8)	0.87	0.52
Mechanical ventilation (d)	0.43 (−0.36–0.89)	6.6	0.07
Anaerobic subgroup			
Vasoactive support (h)*	6.3 (0.96–11.6)	10.3	0.02
* Post hoc subgroup I vs IV*	28.4 (12.2–44.6)	25.1	0.001
Mechanical ventilation (d)*	0.38 (0.09–0.66)	12.9	0.01
* Post hoc subgroup I vs IV*	1.4 (0.62–2.3)	18.1	0.003
Hypoperfusion subgroup			
Vasoactive support (h)*^#^	1.01 (−4.6–6.6)	0.2	0.72
Mechanical ventilation (d)*^#^	0.31 (0.02–0.6)	8.9	0.03

Association of CO_2_-derived variables at admission (H0) with clinical outcomes of interest and association of hemodynamic subgroups with clinical outcomes of interest

*Subgroup 1 is defined as reference

^#^None of the post hoc subgroups comparison was significantly different

Neither the duration of vasoactive support or mechanical ventilation prediction was significantly improved by the addition of CO_2_-derived variables to a model compelling lactate and O_2_ extraction (Supplementary Table 2). Similarly, ΔCCO_2_/AV-DO_2_ did not significantly improve the prediction model when including lactate and the O_2_ extraction for outcome prediction (Supplementary Table 2). In a similar manner, the dynamic evolution of ΔCCO_2_ and ΔCCO_2_/AV-DO_2_ at H6 or H24 was also tested with a model including lactate and O_2_ extraction. Neither the evolution at H6 or H24 of ΔCCO_2_ or ΔCCO_2_/AV-DO_2_ significantly improved outcome prediction, as depicted in Supplementary Table 2.

### Impact of admission hypoperfusion or anaerobic metabolism patterns on outcome

**Fig. 2:**
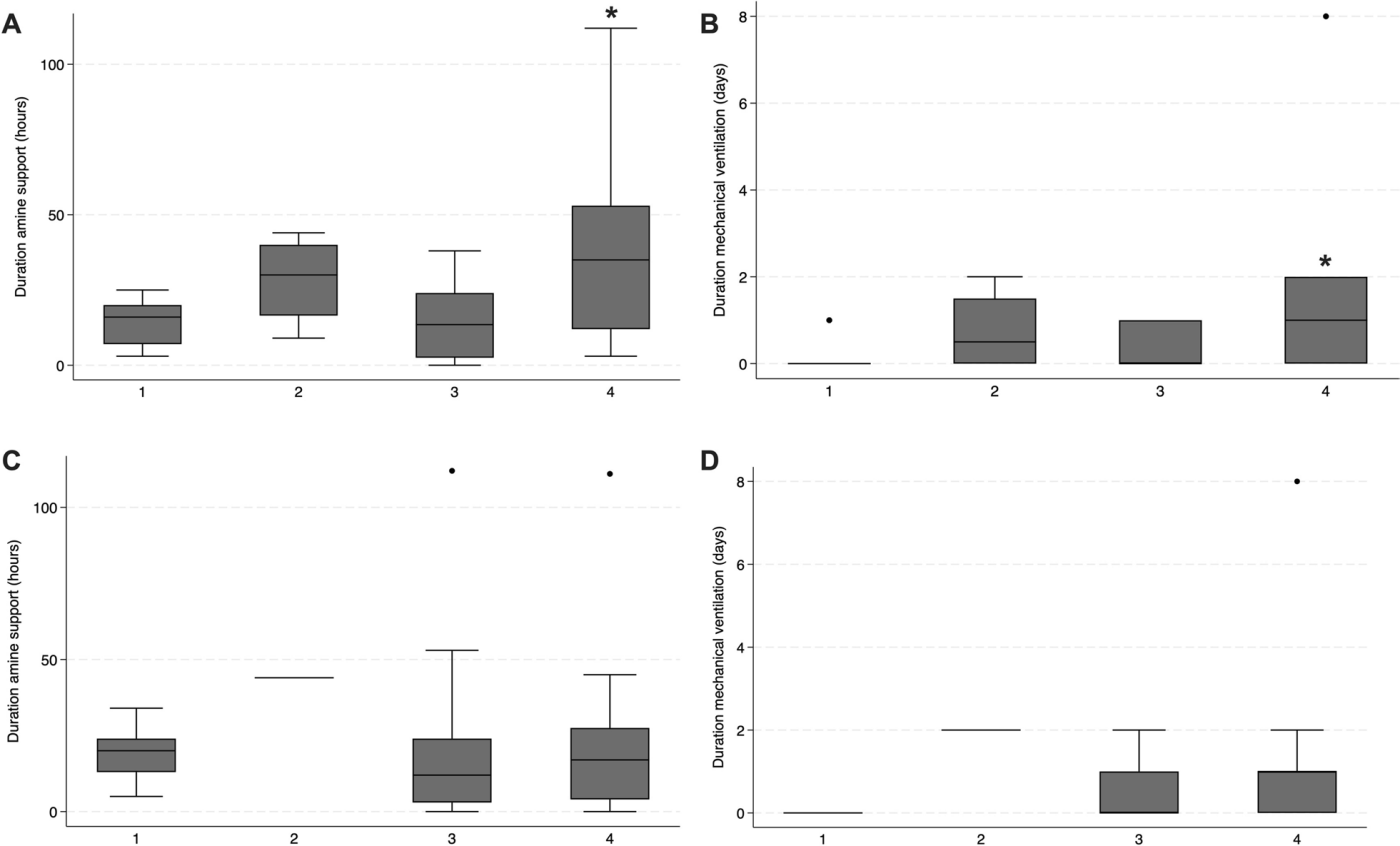
Clinical outcome according to hypoperfusion and anaerobic clinical patterns. Anaerobic subgroups comparison for duration of amine support (**A**) and mechanical ventilation (**B**). Group I, lactate ≤ 2 mmol/L and ΔCCO_2_/AV-DO_2_ ≤ 1.8; group II, lactate > 2 mmol/L and ΔCCO_2_/AV-DO_2_ ≤ 1.8; group III, lactate ≤ 2 mmol/L and ΔCCO_2_/AV-DO_2_ > 1.8; and group IV lactate > 2 mmol/L and ΔCCO2/AV-DO2 > 1.8. *p < 0.05 for subgroup 1 vs. 4. Hypoperfusion subgroups comparison for duration of amine support (**C**), mechanical ventilation support (**D**). Group I, extraction ≤ 30% and ΔCCO_2_ ≤ 6 mL; group II, extraction > 30% and ΔCCO_2_ ≤ 6 mL; group III, extraction ≤ 30% and ΔCCO_2_ > 6 mL; group IV, extraction > 30% and ΔCCO_2_ gradient > 6 mL

Patients were categorized into anaerobic patterns as follows: Subgroup I included 17/51 (33.3%), Subgroup II 4/51 (7.8%), Subgroup III 20/51 (39.2%), and Subgroup IV 10/51 (19.7%). In these anaerobic subgroups, the VIS at admission was 10 (IQR 5–14) for Subgroup I, 19.75 (IQR 13–29) for Subgroup II, 10.5 (IQR 3.75–16.5) for Subgroup III, and 20 (IQR 6–27.5) for Subgroup IV. Clinical outcomes—duration of vasoactive support and mechanical ventilation—differed significantly across the anaerobic subgroups. Post hoc analysis revealed significant differences between Subgroups I and IV for all outcomes (Fig. 2A, B).

For hypoperfusion pattern, the distribution was as follows: Subgroup I 13/51 (25.5%), Subgroup II 1/51 (2%), Subgroup III 21/51 (41.2%), and Subgroup IV 16/51 (31.3%). VIS at admission in these hypoperfusion subgroups was 10 (IQR 5–22) for Subgroup I, 17 (IQR 17–17) for Subgroup II, 10 (IQR 5–22) for Subgroup III, and 14 (IQR 7.25–28.25) for Subgroup IV. In contrast to the anaerobic subgroups, no significant differences were observed among the hypoperfusion subgroups for the duration of amine support or mechanical ventilation (Fig. 2C, D).

## Discussion

This prospective study evaluated the utility of CO_2_-derived variables, specifically ΔCCO_2_ and ΔCCO_2_/AV-DO_2_, as predictors of clinical outcomes in pediatric patients following CPB for CHD surgery. This study provides novel insights into the potential role of CO_2_-derived variables in post-CPB hemodynamic assessment and their integration with O_2_-derived markers to define distinct hemodynamic clinical patterns.

Our findings indicate that ΔCCO_2_ and ΔCCO_2_/AV-DO_2_ were persistently elevated above established adult thresholds (ΔCCO_2_ > 6 mL and ΔCCO_2_/AV-DO_2_ > 1.8) during the first 24 h post-CPB, even in patients with uneventful recoveries. At PICU admission, 72% of patients exhibited ΔCCO_2_ > 6 mL, and 58% had ΔCCO_2_/AV-DO_2_ > 1.8, suggesting that these cutoffs, derived primarily from adult and septic shock populations, may not be applicable to pediatric post-CPB settings [4, 7, 8]. Elevated CO_2_-derived variables have been reported in prior pediatric studies post-CPB, with ΔPCO_2_ (a proxy for ΔCCO_2_) frequently exceeding 6 mmHg [15–17, 28–30]. For instance, Akamatsu et al. noted that 90% of patients had ΔPCO_2_ > 6 mmHg despite low complication rates [28]. Similarly, Taina et al. reported ΔCCO_2_/AV-DO_2_ values between 1.8 and 2 in the post-CPB period [30]. These consistent findings across studies raise questions about the appropriateness of adult-derived CO_2_ thresholds in children, particularly in the context of CPB.

Several factors may contribute to elevated CO_2_-derived variables post-CPB. The post-CPB period is characterized by increased CO_2_ production (VCO_2_) due to repayment of oxygen debt incurred during surgery and active metabolic recovery [31]. Paediatric patients may also exhibit higher baseline VCO_2_ compared to adults, potentially necessitating age-specific thresholds [32, 33]. Additionally, microcirculatory dysfunction, a known feature of the post-CPB state, may impair CO_2_ clearance, leading to elevated venous CO_2_ levels despite adequate cardiac output [34, 35]. This is supported by the hypothesis that CO_2_ diffusion from poorly perfused tissues increases ΔCCO_2_, even in the absence of overt hypoperfusion [27, 34]. These physiological nuances underscore the need to establish pediatric-specific normative values for CO_2_-derived variables post-CPB to enhance their clinical utility.

Regarding outcome prediction, univariate analysis revealed a significant association between admission ΔCCO_2_ and prolonged mechanical ventilation, consistent with findings by Gong et al. [15]. However, no significant association was observed between ΔCCO_2_ or ΔCCO_2_/AV-DO_2_ and the duration of vasoactive support, nor did ΔCCO_2_/AV-DO_2_ independently predict the primary outcome. Moreover, the addition of CO_2_-derived variables to models incorporating lactate and oxygen extraction did not significantly enhance outcome prediction. Discrepancies in the predictive value of ΔCCO_2_ have been noted in prior studies. Rhodes et al. and Insom et al. reported associations between elevated ΔCCO_2_ and adverse outcomes, including mortality, particularly in neonates and patients with univentricular physiology [16, 17]. In contrast, Akamatsu et al. and Taina et al. found no such associations, potentially due to differences in patient populations or outcome definitions [28, 30]. These inconsistencies highlight the need for standardized approaches to population selection and cutoff determination in future studies.

A key contribution of this study is the exploration of hemodynamic clinical patterns defined by combining CO_2_- and O_2_-derived variables. Patients categorized into anaerobic metabolism subgroups based on lactate and ΔCCO_2_/AV-DO_2_ demonstrated significant differences in clinical outcomes. Notably, Subgroup IV (high lactate and high ΔCCO_2_/AV-DO_2_, indicative of severe anaerobic metabolism) exhibited prolonged vasoactive support and mechanical ventilation compared to Subgroup I (normal metabolism). This suggests that integrating ΔCCO_2_/AV-DO_2_ with lactate may improve the identification of patients at risk for adverse outcomes, even when lactate levels are normal. In contrast, hypoperfusion subgroups, defined by oxygen extraction and ΔCCO_2_, did not show significant outcome differences, indicating that anaerobic metabolism may be more clinically relevant in this setting. These findings align with adult septic shock studies, where combining ΔCCO_2_ with lactate or oxygen extraction has refined patient stratification and risk assessment [14, 19, 36]. Castanuela-Sanchez et al. similarly reported that combining ΔCCO_2_ and lactate predicted multiple organ failure post-CPB, supporting the value of multimodal monitoring [37]. However, incorporation of those items in an algorithm-based resuscitation protocol did not change significantly patients’ outcomes in comparison to standard care, underlining the interest in those variables and the need for further studies to specify the role of combining CO_2_- and O_2_-derived variables [38].

The ability of CO_2_-derived variables to complement oxygen-derived markers is particularly promising given the limitations of lactate and oxygen extraction. Lactate levels can be confounded by hyperglycemia, adrenergic support, or impaired clearance, while oxygen extraction may appear falsely normal in conditions like sepsis [5, 6]. CO_2_-derived variables, less susceptible to these confounders, may provide a more robust assessment of hemodynamic status when used in conjunction with traditional markers. To our knowledge, this is the first study to propose anaerobic and hypoperfusion clinical patterns using combined O_2_ and CO_2_ variables in the pediatric post-CPB setting, offering a framework for future investigations into personalized hemodynamic management.

## Limitations

This study has several limitations. Its single-center design and small sample size limit generalizability, and the absence of severe outcomes (e.g., mortality or emergent chest reopening) restricts the evaluation of CO_2_-derived variables in critical scenarios. Incomplete blood gas sampling due to catheter removal may have introduced bias, though the prospective design and systematic sampling mitigate this to some extent. The lack of pediatric-specific normative values for ΔCCO_2_ and ΔCCO_2_/AV-DO_2_ complicates interpretation, particularly in the post-CPB context. Moreover, the impact of a potentially residual shunt may need further studies, but it has not been suggested by other groups. Finally, the study did not establish specific cutoffs for CO_2_-derived variables, limiting immediate clinical applicability.

## Conclusion

This study showed that ΔCCO_2_ holds potential as a predictor of prolonged mechanical ventilation in pediatric patients post-CPB, while ΔCCO_2_/AV-DO_2_ may enhance risk stratification when combined with lactate to define anaerobic metabolism’s. Those hypothesis-generating results underscore the need for further studies investigating multimodal hemodynamic assessment integrating CO_2_- and O_2_-derived variables to offer a promising approach to identifying at-risk patients and tailoring PICU management. Larger, multicenter studies are warranted to validate these findings, define appropriate CO_2_ thresholds, and explore the clinical impact of hemodynamic clinical patterns-guided interventions in pediatric cardiac surgery.

## Supplementary Information

Additional file 1.

## Data Availability

The datasets analyzed during the current study are available from the corresponding author on reasonable request.

## Abbreviations

AV-DO_2_: Arterio-venous oxygen difference
CO_2_: Carbon dioxide
CPB: Cardiopulmonary bypass
CHD: Congenital heart disease
IQR: Interquartile range
PICU: Pediatric intensive care unit
O_2_: Oxygen
CaO_2_: Oxygen content in arterial blood
CvO_2_: Oxygen content in venous
RACHS: Risk adjustment for congenital heart surgery
ΔCCO2/AV-DO_2_: Ratio of ΔCCO_2_ with arterio-venous oxygen difference (AV-DO_2_)
ΔCCO_2_: Veno-arterial CO_2_ content gradient
VIS: Vaso-Inotropic Score
