# Supplementary material for: Veno-arterial CO2 content gradient and veno-arterial CO2 to arterial-venous O2 content ratio for outcome prediction after pediatric cardiac surgery: a prospective study
Source: Intensive Care Med Exp. 2025 Dec 10;13:124. doi: 10.1186/s40635-025-00834-9 (PMC12690030; doi:10.1186/s40635-025-00834-9)
Supplement: Supplementary file 1 — Additional file 1. [file 40635_2025_834_MOESM1_ESM.docx]

**Supplementary files**

**Cousin et al.**

**Supplementary methods**

Central venous (CvCO2) and arterial (CaCO2) CO2 content is calculated as follow:

CCO2 = Plasma CCO2 x [1 - [0.0289 x [Hb] / [[3.352-0.456 x SpO2] x [8.142-pH]]]

Plasma CCO_2_ = 2.226 x S x plasma PCO_2_ x (1+10^pH-pK’^)

S = 0.0307 + [0.00057 x (37-Temp)] + [0.00002 x (37–Temp)^2^]

pK’ = 6.086 + [0.042 x (7.4-pH)] + [(38-Temp) x (0.00472 + (0.00139 x (7.4-pH))]

**Supplementary table 1**

**Outcome prediction in case of dichotomization of VA-CCO2 gradient (cut-off 6 ml) and VA-CCO2/AV-O2 ratio (cut-off 1.8)**

| variable | outcome | R^2^ | Mean difference | 95% CI | p-value |
| --- | --- | --- | --- | --- | --- |
| **At admission** |  |  |  |  |  |
| **Δ**CCO2>6 | Amine | 0.04 | 1.063 | -13.595 to 15.722 | 0.885 |
|  | Mec vent | 4.55 | 0.579 | -0.191 to 1.349 | 0.137 |
|  |  |  |  |  |  |
| **Δ**CCO2/AV-DO2>1.8 | Amine | 2.39 | 7.108 | -6.069 to 20.286 | 0.284 |
|  | Mec vent | 6.65 | 0.637 | -0.056 to 1.330 | 0.071 |
| **At H6 of PICU** |  |  |  |  |  |
| **Δ**CCO2>6 | Amine | 1.29 | 7.381 | -11.560 to 26.322 | 0.437 |
|  | Mec vent | 3.60 | 0.667 | -0.345 to 1.678 | 0.191 |
|  |  |  |  |  |  |
| **Δ**CCO2/AV-DO2>1.8 | Amine | 3.43 | 8.433 | -4.703 to 21.570 | 0.203 |
|  | Mec vent | 3.80 | 0.480 | -0.229 to 1.189 | 0.180 |

Association of CO2-derived variables at admission (H0) and H6 with clinical outcomes of interest: duration of amine support (amine) and duration of mechanical ventilation (mec vent).

**Supplementary table 2**

**Prediction of outcome according to CO2 variables and its adjunction to lactate and O2-extraction**

| variable | outcome | R^2^ | p-value | R^2^ without CO2 variable | R^2^ with CO2 variable | Delta R^2^ | multiple p-value |
| --- | --- | --- | --- | --- | --- | --- | --- |
| **Admission H0** |  |  |  |  |  |  |  |
| **Δ**CCO2 | Amine | 2.87 | 0.240 | 30.64 | 30.64 | 0.00 | 0.972 |
| **Δ**CCO2 | Mec vent | 21.60 | 0.001 | 31.93 | 35.74 | 3.81 | 0.105 |
|  |  |  |  |  |  |  |  |
| **Δ**CCO2/AV-DO2 | Amine | 0.62 | 0.585 | 30.64 | 31.17 | 0.53 | 0.555 |
| **Δ**CCO2/AV-DO2 | Mec vent | 6.21 | 0.081 | 31.93 | 32.77 | 0.84 | 0.453 |
|  |  |  |  |  |  |  |  |
| **Ratio H6-H0** |  |  |  |  |  |  |  |
| **Δ**CCO2 | Amine | 0.33 | 0.697 | 0.28 | 0.39 | 0.12 | 0.821 |
|  | Mec vent | 0.28 | 0.723 | 3.44 | 3.48 | 0.04 | 0.893 |
|  |  |  |  |  |  |  |  |
| **Δ**CCO2/AV-DO2 | Amine | 0.14 | 0.804 | 0.28 | 0.42 | 0.14 | 0.803 |
|  | Mec vent | 1.14 | 0.469 | 3.44 | 4.66 | 1.21 | 0.458 |
|  |  |  |  |  |  |  |  |
| **Ratio H24-H0** |  |  |  |  |  |  |  |
| **Δ**CCO2 | Amine | 1.23 | 0.495 | 32.28 | 32.35 | 0.08 | 0.842 |
|  | Mec vent | 3.60 | 0.241 | 14.20 | 14.68 | 0.48 | 0.654 |
|  |  |  |  |  |  |  |  |
| **Δ**CCO2/AV-DO2 | Amine | 0.00 | 0.992 | 32.28 | 33.84 | 1.56 | 0.362 |
|  | Mec vent | 1.44 | 0.461 | 14.20 | 14.26 | 0.07 | 0.867 |
|  |  |  |  |  |  |  |  |

Impact of addition of CO2-derived variables to predictive models including lactate and O2 extraction for clinical outcome of interest: duration of amine support (amine) and duration of mechanical ventilation (mec vent). Impact assessed for H0, ratio of CO2-derived variable between H6 and H0 and H24 and H0. R^2^ without CO2 variable described prediction of lactate and O2 extraction together and R^2^ with CO2 variable described the R^2^ of the model with the adjunction of either the ΔCCO_2_ or the ΔCCO_2_/AV-DO_2_. Delta R^2^ described the difference between R^2^ with and without CO2-derived variable thus representing the improvement to the model when adding the CO2-derived variable.

**Supplementary figure 1**

**Proportion of normal or elevated VA CCO_2_ and VA CCO_2_/AV O_2_ ratio over the first PICU day**


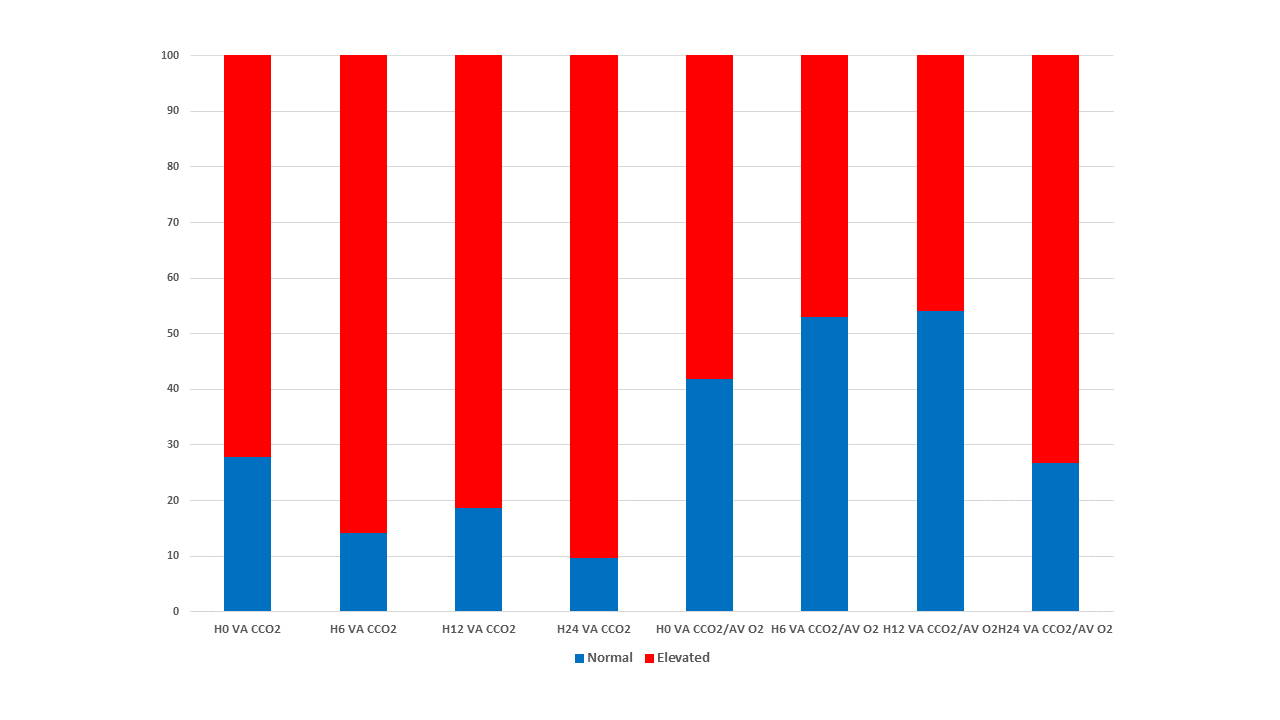


Elevated ΔCCO_2_ and ΔCCO_2_/AV-DO_2_ were defined according to published cut-offs: >6 ml for ΔCCO_2_ and >1.8 for ΔCCO_2_/AV-DO_2_
